# Genome Detective: an automated system for virus identification from high-throughput sequencing data

**DOI:** 10.1093/bioinformatics/bty695

**Published:** 2018-08-16

**Authors:** Michael Vilsker, Yumna Moosa, Sam Nooij, Vagner Fonseca, Yoika Ghysens, Korneel Dumon, Raf Pauwels, Luiz Carlos Alcantara, Ewout Vanden Eynden, Anne-Mieke Vandamme, Koen Deforche, Tulio de Oliveira

**Affiliations:** 1 Emweb bvba, Herent, Belgium; 2 KwaZulu-Natal Research Innovation and Sequencing Platform (KRISP), School of Laboratory Medicine and Medical Sciences, Nelson R Mandela School of Medicine, College of Health Sciences, University of KwaZulu-Natal, Durban, South Africa; 3 The Dutch National Institute for Public Health and the Environment (RIVM), Bilthoven, The Netherlands; 4 Instituto de Ciências Biológicas, Universidade Federal de Minas Gerais, Belo Horizonte, MG, Brazil; 5 Laboratory of Hematology Genetic and computational Biology, Goncalo Moniz Research Center, Oswaldo Cruz Foundation (LHGB/CPqGM/FIOCRUZ), Bahia, Brazil; 6 Laboratório de Flavivírus, IOC, Fundação Oswaldo Cruz; 7 KU Leuven, Department of Microbiology and Immunology, Rega Institute for Medical Research, Clinical and Epidemiological Virology, Leuven, Belgium; 8 Center for Global Health and Tropical Medicine, Unidade de Microbiologia, Instituto de Higiene e Medicina Tropical, Universidade Nova de Lisboa, Lisbon, Portugal

## Abstract

**Summary:**

Genome Detective is an easy to use web-based software application that assembles the genomes of viruses quickly and accurately. The application uses a novel alignment method that constructs genomes by reference-based linking of *de novo* contigs by combining amino-acids and nucleotide scores. The software was optimized using synthetic datasets to represent the great diversity of virus genomes. The application was then validated with next generation sequencing data of hundreds of viruses. User time is minimal and it is limited to the time required to upload the data.

**Availability and implementation:**

Available online: http://www.genomedetective.com/app/typingtool/virus/.

**Supplementary information:**

Supplementary data are available at *Bioinformatics* online.

## 1 Introduction

In the rapidly expanding field of genomics, our ability to produce data far exceeds our capacity to analyze and extract meaningful information. Analysis of viral data is particularly challenging given the high variability of viruses and their deviation from reference genomes, the increasing speed of identification, the continuous emergence of new viruses and the relative scarcity of viral fragments in metagenomic samples (Rose *et al.*, 2016).

The quality of available tools varies and most require specialized computing skills and access to powerful hardware in order to analyze next generation sequencing (NGS) data and/or high-throughput Sanger data. In response to this need, we have developed Genome Detective, a web-based bioinformatics pipeline to accurately and quickly identify, assemble and classify all known viruses present in NGS and Sanger sequencing data.

## 2 Systems and methods

Genome Detective accepts unprocessed paired-end or single reads generated by NGS platforms in FASTQ format and/or processed FASTA sequences. For FASTQ files, low-quality reads are filtered and adapters trimmed with Trimmomatic (Bolger *et al.*, 2014). The quality of the reads is visualized using FastQC (Brown *et al.*, 2017) before and after trimming. Candidate viral reads are identified using the protein-based alignment method DIAMOND (Buchfink *et al.*, 2015). We used the viral subset of the Swissprot UniRef90 protein database, which contains representative clusters of proteins linked to taxonomy IDs, to improve sensitivity and speed. The Swissprot UniRef90 is constantly updated and at the time of the submission of this paper, the viral subset of this database contained 494 134 protein clusters. At the same time, also the NCBI RefSeq database is constantly updated, and at the time of the submission of this paper, the viral subset of this database contained 7560 unique taxonomic IDs. Genome Detective has an automated procedure to download new versions of the reference databases and the current version and the number of viral taxonomy IDs identified are shown on the interface.

The speed and accuracy of Genome Detective was also improved by first sorting short reads into groups, or buckets. Our objective was to run a separate metagenomic *de novo* assembly in each bucket, so all reads of one virus species needed to be assigned to the same bucket. Each bucket is then identified using the taxonomy ID of the lowest common ancestor (LCA) of the hits identified by DIAMOND. However, some reads that represented the same viral species were assigned to buckets at different taxonomic ranks. We solved this problem by either distributing the reads from the node downwards, or collapsing them upwards, by comparing the number of reads identified at each node of the taxonomy tree versus in all descendant nodes. In addition, given that metagenomic studies are accelerating (reviewed in Rose *et al.*, 2016), an increasing number of reference sequences are of novel viruses that have not yet been classified. This causes the LCA taxonomy ID to be unspecific for a number of Uniref clusters, and in the analysis of hits identified by DIAMOND. To avoid these problems, while retaining the sequence themselves, we excluded the taxonomic classification of these viruses in LCA algorithms.

Once all of the reads have been sorted in buckets, each bucket is then *de novo* assembled separately using SPAdes (Bankevich *et al.*, 2012) for single-ended reads or metaSPAdes (Bankevich *et al.*, 2012) for paired-end reads. Blastx and Blastn are used to search for candidate reference sequences against the NCBI RefSeq virus database. Genome Detective combines the results for every detected contig at amino acid and nucleotide (nt) level with by calculating a total score that is a sum of the total nt score plus total amino acid score. We then chose the five best scoring references for each contig to be used during the alignment.

The contigs for each individual species are joined using Advanced Genome Aligner (AGA) (Deforche, 2017), which is a new dynamic programing algorithm. AGA is designed to compute the optimal global alignment considering simultaneously the alignment of all annotated coding sequences of a reference genome. AGA builds further on the optimal alignment algorithms first proposed by Needleman–Wunsch (Smith and Waterman, 1981), Smith–Waterman (Smith and Waterman, 1981) and Gotoh (Gotoh, 1982), by expanding the induction state with additional state parameters. This makes alignments using AGA, and therefore Genome Detective, more sensitive and accurate as both nt and protein scores are taken into account in order to produce a consensus sequence from the *de novo* contigs.

A report is generated, referring to the final contigs and consensus sequences, available as FASTA files. The report also contains detailed information on filtering, assemblage and consensus sequence. Web-based (using the JWt libraries) graphics are available for viral species, genome images, alignment viewer, nt and amino acid similarity measures and read counts. In addition, the user can produce a bam file with BWA (Li and Durbin, 2009) using the reference or *de novo* consensus sequence by selecting the detailed report (Supplementary Fig. S1) and access viral phylogenetic identification tools (de Oliveira *et al.*, 2005) directly from the interface.

## 3 Testing and validation

We first validated Genome Detective using a synthetic virus dataset (NCBI SRA: SRR3458562-SRR3458569), originally prepared to optimize laboratory-based virus extraction procedures, in which viruses were carefully selected to cover the range of naturally occurring diversity (Conceição-Neto *et al.*, 2015). This published dataset also includes carefully validated quantitative results, confirmed with quantitative PCR. Genome Detective identified all of the viruses in the synthetic dataset. We then validated Genome Detective with real clinical datasets. In total, we analyzed 208 datasets, which are available via Sequence Read Archive (SRA) or the European Nucleotide Archive. We then compared our results to the published results and found a >95% concordance, successfully identifying 257 viral species (Table 1 and Supplementary Table). These included single viruses with unsegmented (HIV) and segmented genomes (Influenza A, Rotavirus, MERS) from amplicon-based NGS sequenced as well as unbiased metagenomic datasets (Table 1). Overall, precision, sensitivity and specificity were high, with the exception of 20 metagenomic datasets from human fecal (ERR233412-ERR233431), which had scarce viral reads (Supplementary Table).

**Table 1.** Validation datasets

Publication	PMID	Description	Number of datasets	Expected number of viruses	Assigned number of viruses	Average reconstructed genome size (%)	Number of additional viruses
1	26 559 140	Synthetic virome	8	64	57	92	9
2	Pending (bioRxiv)	Single virus—HIV	14	13	13	93	1
Unpublished	PRJNA434 385 (SRA)	Single virus—HIV	94	94	94	95	15
3	25 609 811	Single virus—RSV	12	12	12	98	1
4	25 056 894	Single virus—norovirus	12	12	12	99	7
5	26 071 329	Single virus—influenza	10	10	10 (80 segments)	94	26
6	24 055 451	Single virus—MERS	14	14	14	94	0
7	28 748 110	Metagenomic—pig fecal	20	20	20 (220 segments)	90	143
8	24 695 106	Metagenomic—human fecal	20	66	25	83	35
	**—**	**—**	**204**	**305**	**257**	**—**	**237**

*Note*: For the validation of Genome Detective (GD) we used 204 datasets from seven studies. This table lists the PMID of the publications, a description of the data, number of datasets, number of viruses originally identified, number of viruses for which GD reconstructed whole genomes (i.e. >80% of the whole genome **and high NT/AA score**) and number of viruses that GD additionally detected (**i.e. <80% of the whole genome or low NT/AA score**). Detailed information such as (SRA files list and full results are seem in Supplementary Material).

We compared our assignment results with IVA (Hunt *et al.*, 2015) and with drVM (Lin and Liao, 2017), which is a new and accurate method for efficient genome assembly of viruses. When the HIV-1 runs were compared with IVA, our web-based application reduced the processing time needed for assembling whole viral genomes by a factor of 10 (10–500-fold) and provided longer and more accurate contigs. In order to compare our results with drVM, we used five datasets (SRR1170797, SRR1106548, DRR049387, SRR062073 and ERR690519). These were the same datasets that the authors of drVM used to compare with three other similar tools, SURPI (Naccache *et al.*, 2014), VIP (Li *et al.*, 2016) and VirusTap (Yamashita *et al.*, 2016). We found that, in general, Genome Detective creates longer, more accurate contigs than drVM, SURPI, VIP and VirusTap. In addition, Genome Detective speed is similar or faster than the four other mentioned tools (Supplementary Material). For example, we assembled a near complete genome (length 8.334 bp) of HIV-1 (SRR1106548) in 430 s, whereas VirusTap identified a 2.896 bp contig in 1.388 s and drVM identified a 3.005 bp segment in 608 s. For the Rotavirus reads (DRR049387), Genome Detective identified all of the 11 segments of Rotavirus A (segment 1–11) in one contig, each covering 97–100% of each segment, whereas rdVM identified only 7 segments from 13 contigs. The time for this run in Genome Detective was 440 versus 464 s of drVM (Lin and Liao, 2017). For Influenza A virus (ERR690519), we identified the same eight segments as drVM in less than half of the time (Supplementary Table S3).

## 4 Discussion

Genome Detective was developed to generate and analyze whole or partial viral genomes directly from NGS reads within minutes. Speed and accuracy were gained by using DIAMOND with a UniProt90 reference dataset to sort viral taxonomy units. The use of DIAMOND and UniRef90 allowed Genome Detective to identify viral short reads at least 1000 times faster than if we used Blastn and the viral nt database of NCBI (Buchfink *et al.*, 2015). Accuracy was also gained by joining contigs with a novel alignment method that uses amino acids and nt scores to create *de novo* contigs. Despite the use of only RefSeq for the identification of virus species, sensitivity and specificity were maintained due to the use of both nt and amino acid similarity scores. We found that for large NGS and metagenomic datasets, Virus Detective substantially reduces computational cost without compromising the quality of the result. However, the construction of *de novo* whole genomes from metagenomic samples depends on the number of reads, the virus genome size and read length. Our pipeline also allows detailed displays of data and results. Furthermore, Genome Detective is linked to our popular virus-specific typing tools (>3 million submissions, de Oliveira *et al.*, 2005), which allow phylogenetic classification below species level. User time is minimal; it is limited to the time required to upload the data. In conclusion, Genome Detective is a web-based pipeline that allows raw NGS data to be assembled into *de novo* complete viral genomes in a fast and accurate manner.

## Funding

Supported by a research Flagship grant from the South African Medical Research Council (MRC-RFA-UFSP-01-2013/UKZN HIVEPI), a Royal Society Newton Advanced Fellowship (TdO), the VIROGENESIS project receives funding from the European Union’s Horizon 2020 Research and Innovation Program (under Grant Agreement no. 634650) and the National Institutes of Health Common Fund, grant number U24HG006941. The content is solely the responsibility of the authors and does not necessarily represent the official views of the National Institutes of Health. We would like to acknowledge the contribution of Annelies Kroneman, Harry Vennema, Roel Standaert, Pieter Libin and Kristof Theys.

*Conflict of Interest*: none declared.

## Supplementary Material

Supplementary Information

Supplementary Table
