# Supplementary material for: Genome Detective: an automated system for virus identification from high-throughput sequencing data
Source: Bioinformatics. 2018 Aug 16;35(5):871–3. doi: 10.1093/bioinformatics/bty695 (PMC6524403; doi:10.1093/bioinformatics/bty695)
Supplement: Supplementary Information [file bty695_supplementary_information.docx]

Supplementary Information:

*Genome Detective: An Accurate, Fast and Automated System for Virus Identification from High-throughput next generation sequencing (NGS) data*

**1) Genome Detective NGS Pipeline Figure**

**2) Genome Detective NGS Reports**

2A) Quality control (QC) and Filtering

2B) Summary table

2C) Detailed table

2D) Detailed Alignment Statistics and Mutations

2E) Mapping short reads to de novo assembled consensus or reference sequence

**3) Accuracy: validation against published results**

**4) Performance: comparison competitor pipelines**

**5) References**

**1) Genome Detective NGS Pipeline**

The diagram below shows the process of assembling next generation sequencing (NGS) short reads into *de novo* consensus sequences (Supplementary figure 1). Black text describes the process; orange text mentions the software applications used. SPAdes is used for single-ended reads and MetaSPADES for paired-end reads. Green lines represent short reads and blue lines the contigs. Bold black lines represent virus reference sequences; grey lines the genomic proteins and green lines represent reference datasets used for phylogenetic typing.

*Supplementary Figure 1: Schematic illustration of Genome Detective NGS Pipeline*

**2 - Genome Detective NGS Reports**

**2A) Quality control (QC) and Filtering:**

The first part of the online report includes quality control (QC), filtering of viral reads and assembly and identification of viral taxonomy units (supplementary figure 1). The size of the input files, the original read length and the trimmed read length are summarized at the beginning of the report. This is followed by a report on the pre-processing and quality control steps, which filter low quality reads and reads that seem to be non-viral. The QC reports of the original submitted reads before and after pre-processing can be extracted from the report. The report also presents the total number of reads assembled and the computational time. A picture of the taxonomy units identified is created. The order of the taxonomy units is based on the number of the reads assembled in the table and in the taxonomy chart, with the largest number reported first.


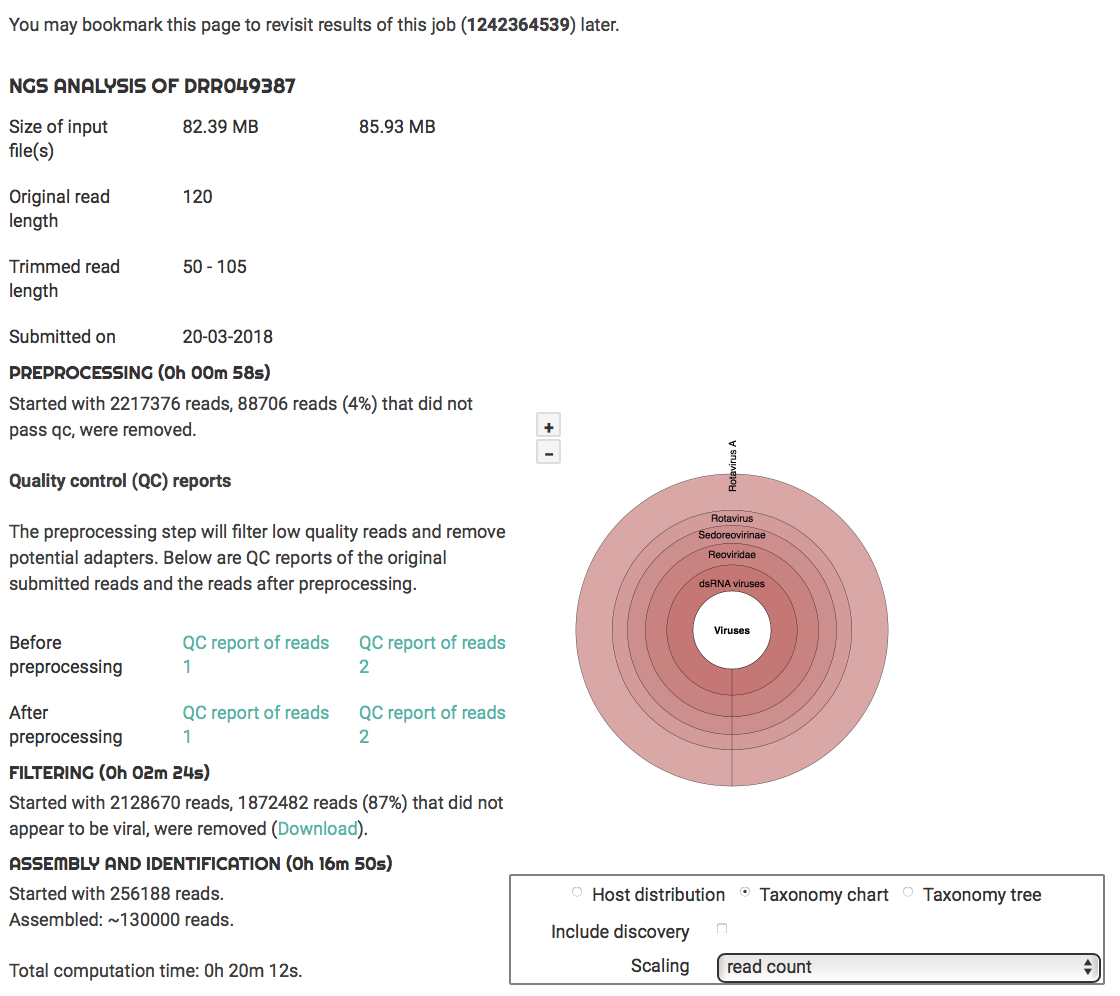


*Supplementary Figure 2: Genome Detective report provides information on the input file, quality control (QC) and filtering of viral reads and de novo assemblage.*

**2B) Summary table:**

The summary table displays information on the assignment, number of *de novo* contigs, estimated number of reads, percentage (%) coverage of the genome, estimated mean depth coverage and nucleotide and amino acid identity (Supplementary table 3). The table also provides a link to download the *de novo* contigs and a link to download the detailed report on each assignment.


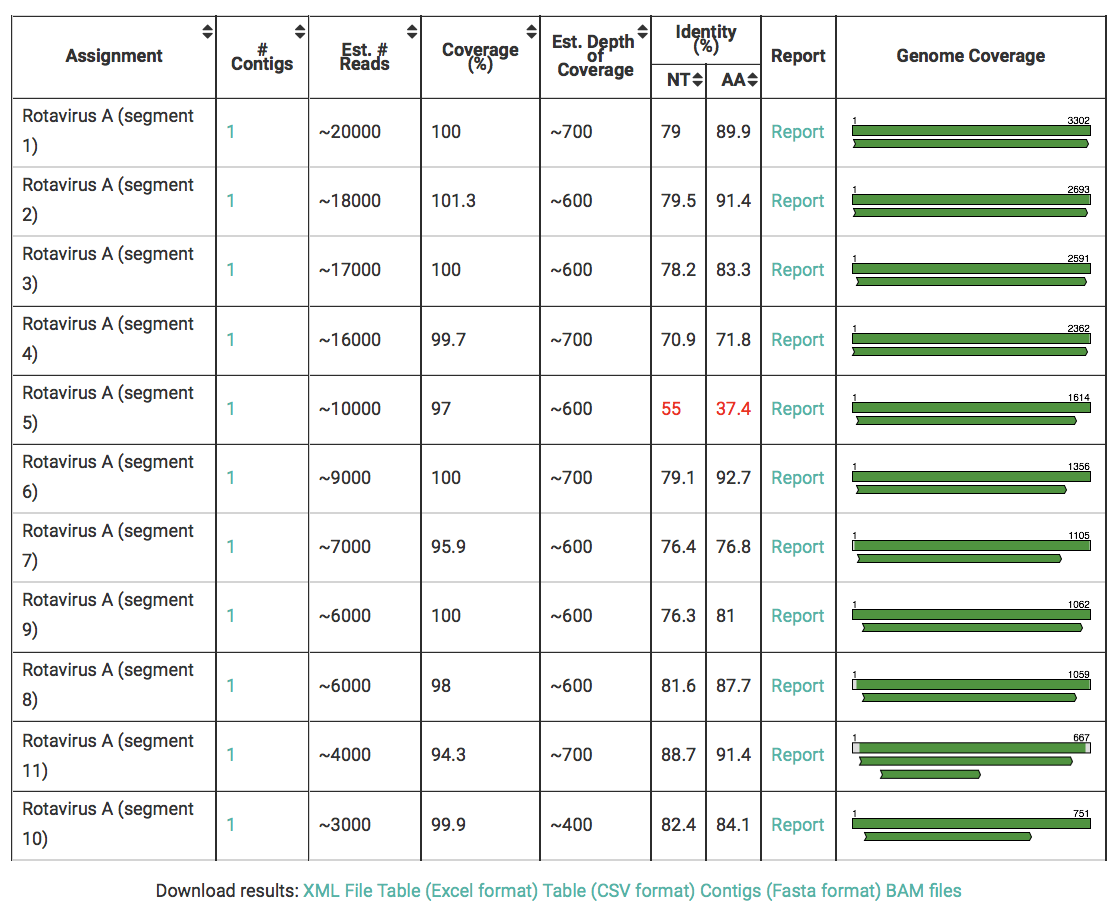


*Supplementary Figure 3: Genome Detective summary table report provides information on the assignment, number of de novo contigs, estimated number of reads, percentage (%) coverage of the genome, estimated mean depth coverage and nucleotide and amino acid identity.*

**2C) Detailed table:**

A detailed table of the assembly is provided for each assignment. This table presents details on the assembly, assignment, alignment, genome region and codon alignment (Supplementary Figure 4).


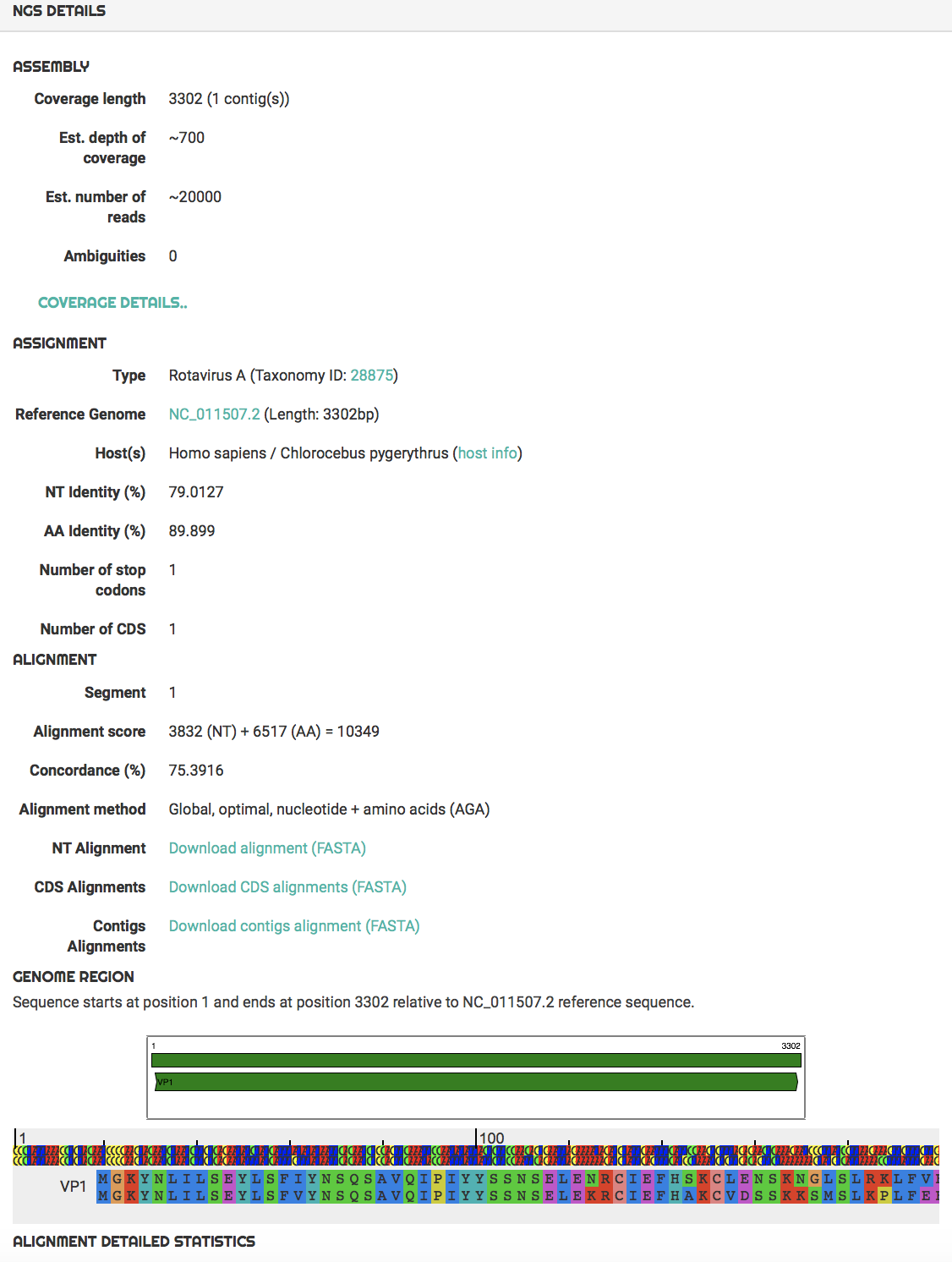
*Supplementary Figure 4:* Genome Detective detailed table report for Rotavirus A de novo whole genome. This report also contains a diagram of the whole genome and the alignment.

**2D) Detailed Alignment Statistics and Mutations:**

The detailed alignment statistics table below shows the nucleotide positions, coding regions and proteins (Supplementary Figure 5). All mutations in the protein are also presented with the following nomenclature (example: I15V, “I” is the wild type amino acid, “15” is the codon position and “V” is the mutant amino acid).


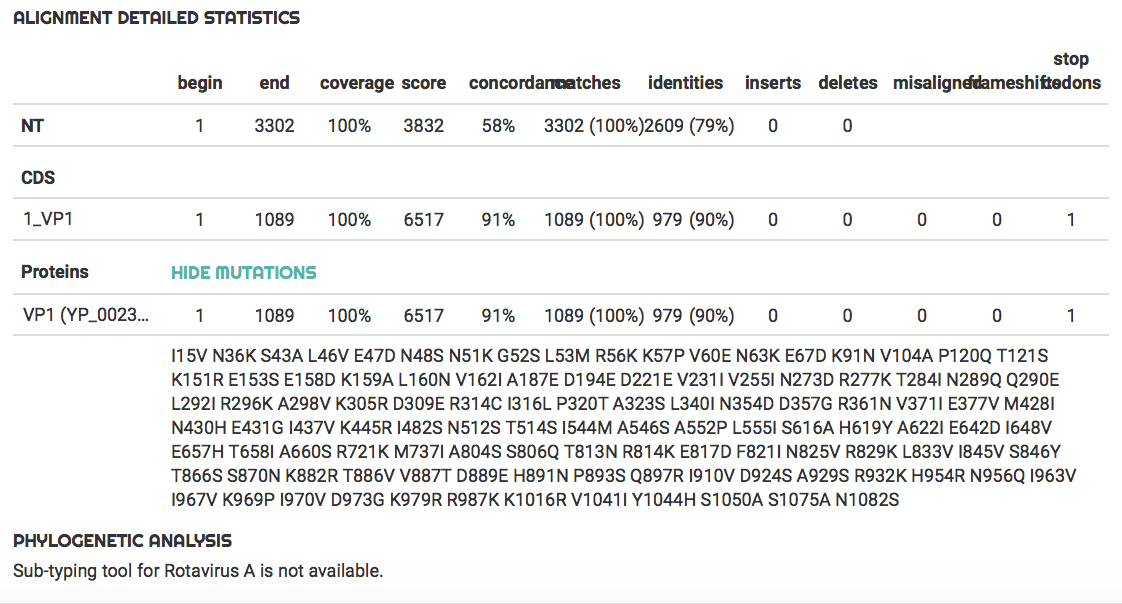


*Supplementary Figure 5: Genome Detective detailed alignment statistics and mutations.*

**2E) Mapping short reads to de novo assembled consensus or reference sequence:**

Genome detective allows the short reads to be mapped to the de novo assembly or to the viral reference sequence from NCBI RefSeq (Supplementary figure 6). Genome detective allows the short read assembly to be downloaded as BAM files and a single nucleotide position (SNP) variant table to be created.

**
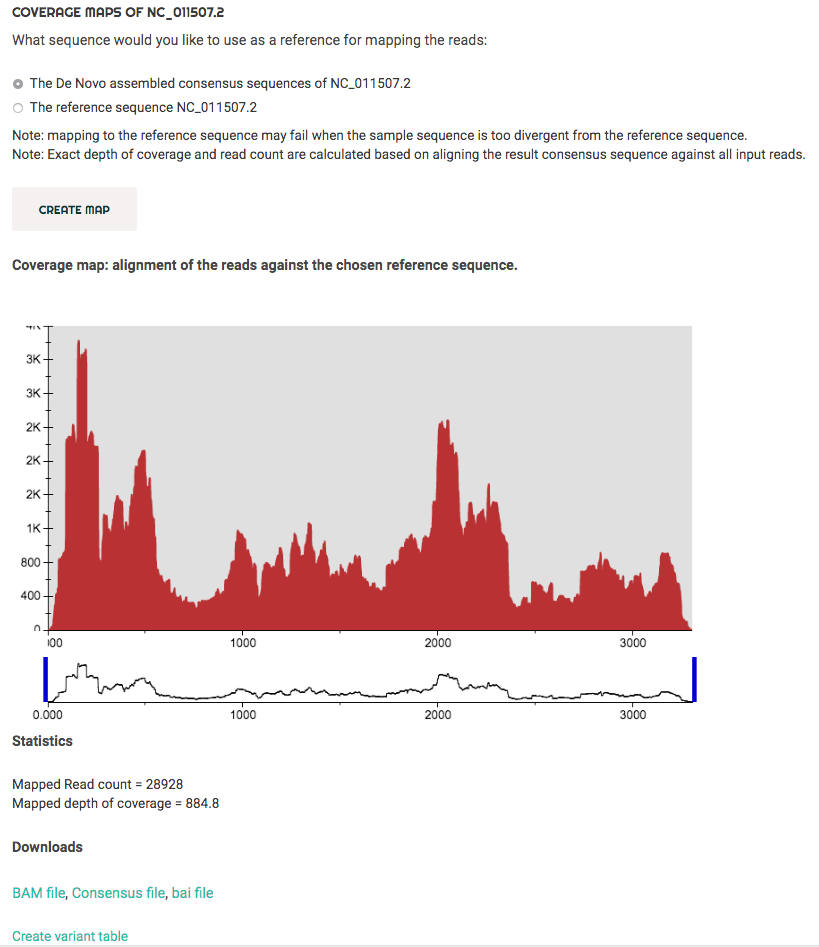
**

*Supplementary Figure 6: Genome Detective coverage maps can be created with the de-novo assembled consensus or with the reference sequence for a given virus. In this example, Rotavirus segment 1 is fully sequenced and assembled.*

**3) Accuracy: validation against published results**

**3A) Detailed results are presented in Supplementary Table 1, which is an Excel table of the supplementary information**

In order to validate the results of Genome Detective, we compared them with the published results from 208 datasets. Summary results are presented in Table 1 of the manuscript. Detailed results are presented in Supplementary Table 1, an Excel table which is part of the supplementary information. Supplementary Table 1 contains detailed information about the 208 datasets used in the validation of Genome Detective. Variables are explained below.

| Dataset name | Run accession number (SRR/ERR) of the dataset |
| --- | --- |
| Reference genome | RefSeq accession number of the closest related virus as identified by Genome Detective |
| Assigned species | Virus species identified by Genome Detective |
| Assigned genus | Virus genus identified by Genome Detective |
| Assignment agreement | Agreement with published result (‘TRUE or FALSE’) |
| Contigs count | The number of contigs generated |
| Coverage % | The percentage of the reference genome covered by the contigs |
| Reads count | The total number of reads used in the generation of the contigs |
| Deep coverage | The average deep coverage of the contigs, as determined by the function:  *read count * read length / contig length,* as estimated by SPAdes |
| N50 (Assembly quality) | A statistical measure of the average length of the contigs. It is widely used to judge assembly quality based on contig lengths |
| **Assignment quality**  The next 5 parameters compare detected virus sequence to the reference genome | |
| AA identity | The percentage amino acid identity in all coding regions of the detected virus  as determined by the function:  *Total number of matching amino acids in coding regions alignment / coding regions alignment length * 100* |
| AA quality | *Amino acid alignment score (see AGA for detailed scoring) / amino acid sequence length* |
| NT identity | The percentage nucleotide identity of the detected virus,  as determined by the function:  *Number of matching nucleotides in alignment / alignment length * 100* |
| NT quality | *Nucleotide alignment score (see AGA for detailed scoring) / nucleotide sequence length* |
| Frame shifts | The number of frame shifts detected in all coding regions of the detected virus |
|  | |
| Stop codon (in CDs) | The total number of stop codons within coding regions of the detected virus sequence |
| Ambiguities (NT) | The number of nucleotide ambiguities in the detected virus sequence |
| Ambiguities (in CDs) | The number of amino acid ambiguities in the detected virus coding regions |
| Assignment quality | “GOOD” indicates that the assignment is considered reliable based on the heuristic of having sufficient nucleotide identity for a sufficient part of the genome, as determined by the function:  *“NT identity” / 100 * “Coverage” + (“Reference length” – “Coverage”) * 0.45 > 0.5 * “Reference length”* |
| **Sample**  General information about the sample | |
| Time | The total analysis time |
| # Reads before QC | The read count in the original dataset |
| # Reads after QC | The number of reads that remain after preprocessing (trimming and removing low quality reads) |
| # Reads after filtering | The number of reads that remain after filtering (alignment against UniRef90 protein database) |
| Read length before QC | The average read length in the original dataset |
|  |  |
| Read length after QC | The average read length after preprocessing |
| Input file (zipped) size | The dataset size |

*Supplementary Table 1 legend for Excel table showing the results of the evaluation of the 208 datasets.*

**3B) Detailed information on the seven datasets presented in Table 1 of the manuscript.**

As previously mentioned in the manuscript and Table 1, 208 datasets from eight studies were used in the validation of Genome Detective. Below, we provide information on the datasets used in the validation process. In addition, we mention the tables and figures from the original publications that were used to validate our results.

**Publication 1 - Virome synthetic datasets.**

We first validated Genome Detective by using synthetic virus datasets originally prepared to optimise laboratory-based virus extraction procedures [Conceição-Neto et al. 2015]. Viruses were carefully selected to cover the range of naturally occurring diversity. This published dataset also included carefully validated quantitative results, confirmed with quantitative PCR. The Supplementary Table S4 in the article described the exact quantity of each virus in every sample. All samples contained Circovirus, Parvovirus, Polyomavirus, Pepino mosaic virus, Rotavirus, Coronavirus, Herpesvirus and Mimivirus in different quantities. Genome Detective identified all of the 64 viruses in the synthetic dataset was reconstructed and for seven the partial genome.

| **Sample** | **Reconstructed** | **Also detected** |
| --- | --- | --- |
| SRR3458562 | Identified 8/8 | Koala retrovirus |
| SRR3458563 | Identified 7/8 (Herpesvirus detected with low genome coverage) | Koala retrovirus |
| SRR3458564 | Identified 7/8 (Mimivirus detected with low genome coverage) | Koala retrovirus and Baboon endogenous virus strain M7 |
| SRR3458565 | Identified 7/8 (Mimivirus detected with low genome coverage) | Koala retrovirus |
| SRR3458566 | Identified 7/8 (Mimivirus detected with low genome coverage) | Koala retrovirus |
| SRR3458567 | Identified 7/8 (Mimivirus detected with low genome coverage) | Koala retrovirus |
| SRR3458568 | Identified 7/8 (Mimivirus detected with low genome coverage) | Gibbon ape leukemia virus |
| SRR3458569 | Identified 7/8 (Mimivirus detected with low genome coverage) | Koala retrovirus |
| Notes:  In all cases, all Rotavirus segments were identified  In several samples, Herpesvirus or Mimivirus were detected with very low genome coverage (< 0.05 %) due to low concentration and large genome size  BLAST search revealed that the additional (false positive) viruses in the “Also detected” column were most likely endogenous viruses that were not in RefSeq, and Genome Detective assigned them to the closest available reference genome | | |

*Supplementary Table 2: Detailed results from synthetic (metagenomic) datasets*

**Publications 2-6 - Single virus amplicon-based datasets – HIV, RSV, Rotavirus, Norovirus, Influenza A and MERs.**

Single virus amplicon-based datasets were used to validate the pipeline with specific viruses [Agoti et al. 2015, Cotton et al. 2014, Rutvisuttinunt et al. 2015, Cotton et al. 2013, de Oliveira et al. 2018]

These included single viruses with segmented genomes (RSV, Rotavirus, Norovirus, Influenza A, MERS) and unsegmented genomes (HIV) from amplicon-based NGS. Because the study design included amplification of the viruses of interest, the reads were expected to cover the amplified fragments. In the single virus datasets, we reconstructed the genomes of all of the amplified virus fragments with high accuracy. Furthermore, Genome Detective produced longer contigs than most of the competitor pipelines.

**Publication 7 – Human and pig samples for Rotavirus.**

This study analyzed human and pig samples for Rotavirus (RV). They used a primer-independent, agnostic, deep sequencing approach [Phan et al. 2016].

***Publication 8 – Metagenomic dataset***

The study [Cotten et al. 2014] analyzed 20 metagenomic datasets and they provided a heat map (Fig 6 of the article). Genome Detective identified the 66 virus species with more then 10 reads as the original study did.

**4) Performance: comparison with competitor pipelines**

In order to compare Genome Detective’s performance with other pipelines, we used four published datasets: SRR1170797, SRR1106548, DRR049387 and ERR690519. These are four of the five datasets that the authors of drVM [Lin & Liao et al. 2017] used to compare with three other tools, SURPI [Naccache et al. 2014], VIP [Li et al. 2016] and VirusTap [Yamashita et al. 2016] (Supplementary Table 3).

We found that, in general, Genome Detective created longer, more accurate contigs than drVM, SURPI, VIP and VirusTap. In addition, Genome Detective was faster than the four other tools. For example, in the HIV-1 dataset (SRR1106548), Genome Detective assembled a near complete genome (8,334 of 9,181 bps) in 430 sec, whereas drVM identified a 3,055 bp contig in 608 sec. Both Genome Detective and drVM also identified Torque teno virus. For the Rotavirus dataset (DRR049387), Genome Detective identified all of the 11 segments of Rotavirus A (segment 1 to 11), each with one contig covering 97-100% of the segment, whereas drVM identified 13 contigs covering only seven segments. The time for this run in Genome Detective was 440 seconds whereas if took 464 seconds in drVM [Lin & Liao 2017]. For Influenza A virus (ERR690519), we identified the same eight segments as drVM in less than half the time. Supplementary table 3 is adapted from the drVM paper, which compared performance between drVM, SURPI, VIP and VirusTAP. We were unable to locate the fifth dataset (SRR062073) in the public databases. (see - https://www.ebi.ac.uk/ena/data/view/SRR062073&display=html).

| **Target virus (run accession)** | **Read bases (Mbp)** | | **Genome Detective** | **drVM** | **SURPI (comprehensive)** | **VIP (sense)** | **VirusTAP** |
| --- | --- | --- | --- | --- | --- | --- | --- |
| Bovine viral diarrhea virus (SRR1170797) | 12.5 | Run time | **71 s** | 149s | 52 365 s | 1 683 s | 71 s |
|  |  | Result | **11 906 bp** | **12 224 bp** | 262 bp | 9078 bp | 353 bp |
| Human immunodeficiency virus (SRR1106548) | 600.9 | Run time | **430 s** | 598 s | 32 604 s | 25 049 s | 1 388 s |
|  |  | Result | **8 334 bp** | 3 055 bp | 799 bp | 4632 bp | 2 896 bp |
| Human rotavirus A (DRR049387) | 266.1 | Run time | **440 s** | 464 s | 59 510 s | 6259 s | 925 s |
|  |  | Result | **11 contigs (11 segments, 99% complete)** | 13 contigs (7 segments) | 13 contigs | 41 377 reads | 11 contigs |
| Influenza A virus (ERR690519) | 3300 | Run time | **1 423 s** | 12 697 s | 16 997 s | 86 001 s | 4 504 s |
|  |  | Result | **8 contigs (8 segments, 99% complete)** | **8 segments** | 11 contigs | 2673 reads | 34 contigs |

*Supplementary Table 3: Performance Comparison with other pipelines. We analyzed the Genome Detective data for the same datasets analyzed by drVM. This table was adapted from Lin & Liao 2017 (https://www.ncbi.nlm.nih.gov/pmc/articles/PMC5466706/table/tbl3/.) Genome Detective’s analysis was executed on a quad-core CPU with 64 GB RAM. The drVM, SURPI and VIP analyses were executed on a quad-core CPU with 128 GB RAM, and the VirusTAP analyses executed on a 120-core CPU with 1 TB RAM.*

**5) References:**

Agoti, C. N., Otieno, J. R., Munywoki, P. K., Mwihuri, A. G., Cane, P. A., Nokes, D. J., … Cotten, M. (2015). Local Evolutionary Patterns of Human Respiratory Syncytial Virus Derived from Whole-Genome Sequencing. *Journal of Virology*, *89*(7), 3444–3454. <http://doi.org/10.1128/JVI.03391-14>

Conceição-Neto, N., Zeller, M., Lefrère, H., De Bruyn, P., Beller, L., Deboutte, W., … Matthijnssens, J. (2015). Modular approach to customise sample preparation procedures for viral metagenomics: a reproducible protocol for virome analysis. *Scientific Reports*, *5*, 16532. Retrieved from <http://dx.doi.org/10.1038/>

Cotten, M., Petrova, V., Phan, M. V. T., Rabaa, M. A., Watson, S. J., Ong, S. H., … Baker, S. (2014). Deep Sequencing of Norovirus Genomes Defines Evolutionary Patterns in an Urban Tropical Setting. *Journal of Virology*, *88*(19), 11056–11069. <http://doi.org/10.1128/JVI.01333-14>

Cotten, M., Oude Munnink, B., Canuti, M., Deijs, M., Watson, S. J., Kellam, P., & van der Hoek, L. (2014). Full Genome Virus Detection in Fecal Samples Using Sensitive Nucleic Acid Preparation, Deep Sequencing, and a Novel Iterative Sequence Classification Algorithm. PLoS ONE, 9(4), e93269. http://doi.org/10.1371/journal.pone.0093269

Cotten M, Watson SJ, Kellam P, Al-Rabeeah AA, Makhdoom HQ, Assiri A, … Memish ZA. (2013). Transmission and evolution of the Middle East respiratory syndrome coronavirus in Saudi Arabia: a descriptive genomic study. Lancet 382(9909):1993-2002. doi: 10.1016/S0140-6736(13)61887-5.

de Oliveira T, Giandhari J. HIV-1 – umpublished, but data deposited at SRA - PRJNA434385.

Lin, H.-H., & Liao, Y.-C. (2017). drVM: a new tool for efficient genome assembly of known eukaryotic viruses from metagenomes. *GigaScience*, *6*(2), 1–10. Retrieved from <http://dx.doi.org/10.1093/>

Phan, M. V. T., Anh, P. H., Cuong, N. Van, Munnink, B. B. O., van der Hoek, L., … My, P. T. (2016). Unbiased whole-genome deep sequencing of human and porcine stool samples reveals circulation of multiple groups of rotaviruses and a putative zoonotic infection. *Virus Evolution*, *2*(2), vew027-vew027. Retrieved from <http://dx.doi.org/10.1093/ve/>

Rutvisuttinunt, W., Chinnawirotpisan, P., Thaisomboonsuk, B., Rodpradit, P., Ajariyakhajorn, C., Manasatienkij, W., … Fernandez, S. (2017). Viral subpopulation diversity in influenza virus isolates compared to clinical specimens. *Journal of Clinical Virology*, *68*, 16–23. <http://doi.org/10.1016/j.jcv>[.](http://doi.org/10.1016/j.jcv.2015.04.010)

Wymant, C., Blanquart, F., Gall, A., Bakker, M., Bezemer, D., Croucher, N. J., … Fraser, C. (2016). Easy and Accurate Reconstruction of Whole HIV Genomes from Short-Read Sequence Data. *bioRxiv*. Retrieved from [http://biorxiv.org/content/](http://biorxiv.org/content/early/2016/12/09/092916.abstract)
